# Cardiovascular magnetic resonance angiography for the detection of anomalous coronary arteries: a case report

**DOI:** 10.4076/1757-1626-2-6835

**Published:** 2009-09-15

**Authors:** Sofia Chatzidou, Sotiris C Plastiras, Stelios Rokas

**Affiliations:** 1 Department of Clinical Therapeutics, Interventional Cardiology Unit, University of Athens Medical School, "Alexandra Hospital", 80 Vasilissis Sofias av & Lourou St, Athens, 11528, Greece

## Abstract

Coronary artery anomalies occur in approximately 0.3% to 0.8% of the population, and include morphological variants of origin, course, or termination. Detection of these types of anomalously originating coronary arteries is crucial for therapeutic intervention.

## Introduction

Coronary magnetic resonance angiography (CMRA) appears to be a useful modality in the imaging of the proximal course of anomalous coronary arteries. We report a case of an anterior course of the LMCA in relation to the pulmonary trunk, highlighting the usefulness of cardiac MRA in detecting the anomalously originated coronary arteries.

## Case presentation

**Figure 1:**
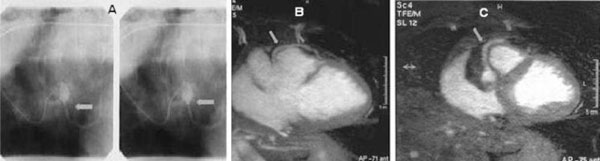
**(A) Conventional x-ray angiography showing an anomalous originating left main coronary artery (LMCA, arrow) from the right aortic sinus**. **(B, C)** Transverse MR angiographic images showing an anterior course of the LMCA in relation to the pulmonary trunk (arrow).

We report on the case of a 55-year old man (Caucasian, Athens, Greece) with a recent episode of syncope. He had a history of exercise related palpitations within the past 3 months, but no ischemic heart disease or other cardiomyopathy and/or thyroid disease. Standard 12-lead electrocardiogram and two-dimensional echocardiography were normal. A run of non sustained monomorphic ventricular tachycardia was recorded on Holter monitoring, and during recovery after maximal exercise stress test. Thallium-201 myocardial perfusion imaging showed a minor perfusion defect in the anterior wall. On coronary angiography the left main coronary artery (LMCA), the left anterior descending (LAD), the left circumflex (LCX), and the right coronary artery (RCA) had no stenoses, but both LAD and RCA originated from the right sinus of Valsava (RSOV, Figure 1A). On further work-up, a coronary magnetic resonance angiography (CMRA) was performed. CMRA revealed an anterior course of the LMCA in relation to the pulmonary trunk (Figures 1B and 1C). The patient was scheduled for coronary artery bypass grafting (CABG). A left internal mammary artery conduit was successfully grafted to the LAD and a vein graft to the LCX.

## Conclusion

Coronary artery anomalies occur in approximately 0.3% to 0.8% of the population, and include morphological variants of origin, course, or termination. Not all anomalies will have sequelae that serious. The pattern with proximal coronary artery segments crossing between the aorta and the pulmonary trunk has been reported to be potentially lethal [1]. The treatment of the anomaly originating LMCA from the RSOV is controversial and mainly based on observations [2]. Basically the three benign forms (Septal course along the floor of the right ventricle, anterior right ventricle free wall course, Retro-aortic course) do not require specific interventions, while the intra-arterially crossing LMCA may warrant surgical repositioning, especially if associated with objective evidence of ischemia [3].

Detection of these types of anomalously originating coronary arteries is important for clinical intervention. With the current standard technique, ie, conventional coronary angiography, the exact proximal course of anomalous coronary arteries may be difficult to determine [4]. Misdiagnosis has been reported to occur in up to 50% of patients. CMRA is a novel noninvasive technique that has proved to be accurate in the imaging of proximal coronary anatomy [5]. CMRA appears to be a very useful adjunct coronary angiography and, with confirmation of our results in larger series, might even be considered a new gold standard in the imaging of the proximal course of anomalous coronary arteries. In this particular patient group, CMRA allowed precise delineation, monitoring its progression, and potentially evaluating the effects of therapeutic intervention.

## Consent

Written informed consent was obtained from the patient for publication of the manuscript and figures. A copy of the written consent is available for review by the Editor-in-Chief of this journal. The ethnic origin of the patient described in the present report is Greece.

## Competing interests

The authors declare that they have no competing interests.

## Authors' contributions

The case-report was written by SC and SP and extensively reviewed by SR.
